# Multiband multi-echo imaging of simultaneous oxygenation and flow timeseries for resting state connectivity

**DOI:** 10.1371/journal.pone.0169253

**Published:** 2017-03-02

**Authors:** Alexander D. Cohen, Andrew S. Nencka, R. Marc Lebel, Yang Wang

**Affiliations:** 1 Department of Radiology, Medical College of Wisconsin, Milwaukee, Wisconsin, United States of America; 2 GE Healthcare, Calgary, Alberta, Canada; Institute of Psychology, Chinese Academy of Sciences, CHINA

## Abstract

A novel sequence has been introduced that combines multiband imaging with a multi-echo acquisition for simultaneous high spatial resolution pseudo-continuous arterial spin labeling (ASL) and blood-oxygenation-level dependent (BOLD) echo-planar imaging (MBME ASL/BOLD). Resting-state connectivity in healthy adult subjects was assessed using this sequence. Four echoes were acquired with a multiband acceleration of four, in order to increase spatial resolution, shorten repetition time, and reduce slice-timing effects on the ASL signal. In addition, by acquiring four echoes, advanced multi-echo independent component analysis (ME-ICA) denoising could be employed to increase the signal-to-noise ratio (SNR) and BOLD sensitivity. Seed-based and dual-regression approaches were utilized to analyze functional connectivity. Cerebral blood flow (CBF) and BOLD coupling was also evaluated by correlating the perfusion-weighted timeseries with the BOLD timeseries. These metrics were compared between single echo (E2), multi-echo combined (MEC), multi-echo combined and denoised (MECDN), and perfusion-weighted (PW) timeseries. Temporal SNR increased for the MECDN data compared to the MEC and E2 data. Connectivity also increased, in terms of correlation strength and network size, for the MECDN compared to the MEC and E2 datasets. CBF and BOLD coupling was increased in major resting-state networks, and that correlation was strongest for the MECDN datasets. These results indicate our novel MBME ASL/BOLD sequence, which collects simultaneous high-resolution ASL/BOLD data, could be a powerful tool for detecting functional connectivity and dynamic neurovascular coupling during the resting state. The collection of more than two echoes facilitates the use of ME-ICA denoising to greatly improve the quality of resting state functional connectivity MRI.

## Introduction

The impact and applications of resting-state functional connectivity magnetic resonance imaging (rs-fcMRI) continue to grow. Developed in 1995 [1], rs-fcMRI is collected while a subject is not performing a task. rs-fcMRI uses correlations between low-frequency fluctuations in the signal to extract connected brain networks. Most rs-fcMRI studies rely on blood oxygenation-level dependent (BOLD) contrast, which measures magnetic susceptibility changes caused by variations in blood oxygenation. However, BOLD contrast is sensitive to cerebral blood flow (CBF) and cerebral blood volume (CBV), in addition to blood oxygenation [2]. Recently, arterial spin labeling (ASL), which measures CBF changes directly by magnetically tagging blood flowing into the brain, has been used for rs-fcMRI [3–6].

Evaluated separately, BOLD and ASL contrasts each have several advantages and disadvantages for rs-fcMRI. The BOLD signal can be acquired with high temporal and spatial resolution, resulting in images with high temporal signal-to-noise ratios (tSNR) and sensitivity; however, the BOLD signal is an indirect measure of neuronal activity and results from the combination of a number of factors including CBF, CBV, and oxygen consumption [2, 5, 7]. Thus, inferring neuronal underpinnings directly from the BOLD signal is not straightforward. Furthermore, the BOLD signal is sensitive to physiological factors including motion, respiration, and cardiac influences [8]. The BOLD signal also has limited spatial specificity due in part to its susceptibility to draining veins [9–11]. Conversely, the ASL signal is derived from capillaries and provides a more direct, spatially specific measure of neuronal activity. ASL can also be used to derive quantitative CBF, which is directly related to brain physiology. ASL, however, requires a tagging module to label inflowing blood and a post-labeling delay (PLD) to allow tagged blood to flow into the brain. For pseudo-continuous ASL (pCASL), the recommended approach for ASL imaging [12], the suggested tagging time and PLD are each more than 1.5 s, resulting in long repetition times (TRs). Additionally, the total readout times of ASL acquisitions are severely limited by the short T1-relaxation of the tagged blood. This, in turn, reduces the signal-to-noise ratio (SNR) and restricts the image resolution and total number of slices that can be acquired.

To address some of these issues, several sequences have been developed to obtain ASL and BOLD contrast simultaneously by collecting ASL- and BOLD-sensitive echoes in one acquisition [13–17]. These sequences have several applications. First, the coupling between CBF and BOLD has been evaluated to determine the contributions of CBF to the BOLD response [15, 16]. These studies have found functional connectivity to be positively correlated with CBF/BOLD coupling within functional networks. Second, cerebrovascular reactivity (CVR) measurements, which measure the ability of blood vessels to dilate [18], have been obtained [19]. BOLD- and CBF-derived CVR measurements have been shown to be complimentary [20], making simultaneous ASL/BOLD approaches attractive. Finally, simultaneous ASL/BOLD scans have been used to calibrate the BOLD signal [21, 22] and to compute the cerebral metabolic rate of oxygen consumption (CMRO_2_) [23]. CMRO_2_ fluctuations have also been used to generate resting-state brain networks [24]. However, these sequences still necessitate long TRs and a limited number of slices.

Recently developed multiband (MB), or simultaneous multi-slice (SMS) imaging, where multiple slices are excited and acquired simultaneously, can be used to increase spatial and/or temporal resolution [25, 26]. MB imaging has been developed and validated for functional magnetic resonance imaging (fMRI) [27] and rs-fcMRI [26, 28]. MB imaging also has been combined with ASL to acquire high-resolution ASL images [29–31]. These studies only evaluated static CBF measurements, not dynamic CBF timeseries necessary for rs-fcMRI studies. Because the majority of the TR in ASL consists of tagging and PLD modules, the major advantage of MB-ASL is that interslice labeling delay times and total readout times can be reduced. This leads to increased SNR and more accurate CBF estimations and increases the number of slices that can be acquired [29–31].

Emerging research has shown multi-echo (ME) echo-planar imaging (EPI) techniques have the ability to increase the sensitivity of BOLD acquisitions [32–37]. These approaches acquire several echoes in one excitation. Echoes can then be combined by weighting each echo by the voxelwise T2* [32, 34, 38]. This weighting approach takes advantage of the fact that BOLD contrast is maximized when the echo time (TE) is equal to T2* in the tissue. Since T2* varies by tissue type, different weightings are used for different brain regions. BOLD sensitivity can be further increased by applying ME denoising procedures to the data. One such technique, ME independent component analysis (ME-ICA), can automatically separate BOLD and non-BOLD echoes [39–42]. This technique uses ICA to identify signal components representing the most variance in the data. These components then get classified as either BOLD or non-BOLD based on whether or not their amplitudes are linearly dependent on TE, respectively. The non-BOLD components are then removed from the data via linear regression. These studies have shown ME-ICA increases individual and group level functional connectivity [39, 40], can separate slow BOLD from non-BOLD drifts [37], and can also be used to increase task-based sensitivity [42]. ME-ICA has also been evaluated for MB BOLD data showing improved performance over SB acquisitions [43]. To date, simultaneous ASL/BOLD techniques acquire only two echoes and do not take advantage of the sensitivity gains of acquiring additional echoes.

In this study, four techniques—MB, ME, ASL, and BOLD—are combined to create a single sequence. Using this sequence, high resolution, whole-brain simultaneous ASL/BOLD data were acquired to evaluate resting-state connectivity.

## Methods

### MBME ASL/BOLD sequence

**Fig 1:**
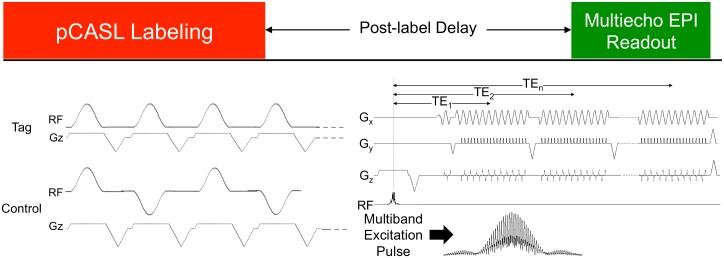
MBME ASL/BOLD pulse sequence design. The sequence consists of a pCASL labeling train, followed by a post-label delay, and a multi-echo EPI readout. The phase is rewound to the start of k-space after each echo and the next echo is acquired. Multiband imaging was also utilized by inserting a multiband excitation pulse in place of the single band pulse. Finally, blipped-CAIPI was employed to reduce g-factor penalties associated with MB imaging.

Fig 1 shows a sequence diagram for the MBME ASL/BOLD sequence. The general sequence design consists of an unbalanced pCASL tagging module [44, 45], followed by a PLD period. This is followed by a MB excitation with a ME EPI readout. Blipped-controlled aliasing in parallel imaging (blipped-CAIPI) [28] was employed to reduce g-factor noise amplification caused by the slice-unaliasing in MB imaging by incorporating z-gradient blips. This technique shifts individual simultaneously excited slices in image space by fractions of the field of view to reduce aliasing of the slices.

At the start of each MBME ASL/BOLD acquisition, calibration repetitions were acquired using a radiofrequency (RF) phase-cycling approach [46]. Varying constant phase shifts were added to subsequent MB RF pulses to allow the slices to be unaliased using a discrete Fourier transform. For example, to unalias four slices, four repetitions are required. This method ensures identical geometric distortions between the calibration image and the actual acquisition. For acquisitions incorporating in-plane acceleration (R), such as the ME approach used here, a multishot technique was applied. For each constant phase, R interleaves were collected and combined to fill 2D k-space. This allowed 2D k-space to be fully sampled while keeping the same distortion as the functional repetitions. The last repetition in each acquisition was acquired with no tagging to obtain an M0 image, used to normalize the subtracted perfusion-weighted (PW) maps for CBF quantification.

Each echo in the ME acquisition was obtained consecutively with a single excitation. The user prescribed the timings of the first and second echoes. Subsequent TEs were set at the minimum time following the previous echoes. Of note, the readout was identical for calibration and functional repetitions. The only difference was the varying RF phase for the calibration repetitions and the addition of blipped-CAIPI for the functional repetitions. Navigator echoes were acquired at the beginning of each shot by sampling the center of k-space several times in the positive and negative frequency encode directions. These echoes were used to correct for Nyquist ghosting in each shot and repetition.

### Subjects

This study was approved by the Medical College of Wisconsin/Froedtert Hospital Institutional Review Board, and all subjects provided written informed consent before participating. Seven healthy adult volunteers (four male, three female, mean age = 35.0 +/- 13.6 years, age range 23–58 years) were recruited for this study. All subjects were right-handed. Subjects were asked to refrain from intake of caffeine before the MRI exam.

### Imaging

Imaging was performed on a GE 3 Tesla MR750 system with a body transmit coil and 32-channel NOVA receive head coil. High-resolution anatomical images were collected for co-registration with the functional images. These included a T1-weighted magnetization-prepared rapid acquisition with gradient echo (MPRAGE) with the following parameters: TR/TE = 7.6/3.0 ms, FA = 8°, FOV = 256 mm, 1×1×1 mm^3^ resolution, BW = 62.5 kHz, and TI = 900 ms. An additional T2-weighted image was also acquired using a CUBE sequence with the following parameters: TR/TE = 2500/63.6 ms, FA = 90°, FOV = 256 mm, 1×1×1mm^3^ resolution, and BW = 125 kHz.

### Resting-state scan protocol

Each subject underwent one resting-state MBME ASL/BOLD scan, which utilized an unbalanced pCASL labeling scheme with labeling time = 1.5 s and PLD = 1.0 s. Calibration volumes were acquired at the start, and as part, of each MBME ASL/BOLD scan. A partial k-space acquisition was employed with 20 overscan lines. To keep the later TEs within reasonable ranges and reduce total readout time, in-plane acceleration was employed with R = 2. Additional parameters for the MBME ASL/BOLD run were as follows: number of echoes = 4, TE = 9.1, 25, 39.6, 54.3 ms, TR = 3.5 s, MB-factor = 4, number of excitations = 9 (total slices = 9×4 = 36), FOV = 240 mm, resolution = 3×3×3 mm^3^, FA = 90°, RF pulse width = 6400 ms. Scans lasted 644 seconds resulting in 167 acquisitions excluding calibration reps. Subjects were instructed to lie awake with their eyes closed.

### Reconstruction

All image reconstruction was performed in Matlab (MathWorks, Inc.). First, Nyquist ghosting correction was performed using navigator echoes collected at the beginning of each excitation. Next, the echoes were separated.

Calibration repetitions were unaliased using a discreet Fourier transform. In this study, two calibration volumes were acquired and the second one was used as the calibration image for slice and in-plane unaliasing. A slice-GRAPPA algorithm [28] was implemented for MB unaliasing and applied separately for each echo. This technique is described in detail elsewhere [28]. To unalias in-plane acceleration, a traditional 1D-GRAPPA approach [47] was used following the slice-unaliasing procedure. Finally, coils were combined using a sum of squares approach, and partial k-space was reconstructed using a homodyne technique [48].

### Preprocessing

**Fig 2:**
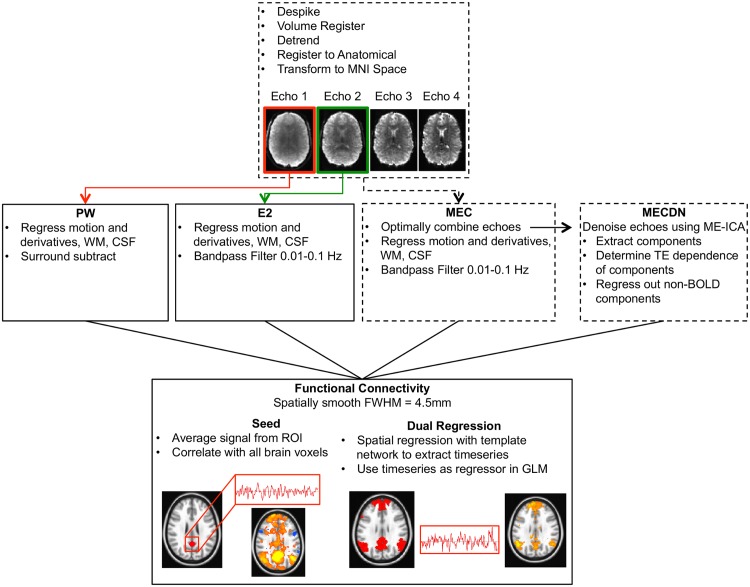
Schematic showing the resting state functional connectivity pipeline for the ASL and BOLD echoes. The first and second echoes were processed separately to yield the PW and E2 data respectively. Echoes were also combined using a T2*-weighted approach to generate the MEC dataset. This dataset was further denoised using ME-ICA resulting in the MECDN dataset. Each echo was despiked, volume registered using the transformation matrix from the first echo, detrended, registered to the anatomical image, and transformed to MNI space. Additional preprocessing steps differ for each dataset and are shown in Row 2. For all datasets, after preprocessing, functional connectivity was assessed with seed-based and dual-regression approaches (Row 3).

The rs-fcMRI data processing pipeline is shown in Fig 2. Prior to ASL processing and echo combination and denoising, preprocessing was performed on each echo separately using AFNI (https://afni.nimh.nih.gov/afni) and FSL (http://fsl.fmrib.ox.ac.uk/fsl/fslwiki). This procedure included despiking, volume registration, detrending with a 3^rd^ order polynomial, and skull-stripping of the data. For the volume registration, motion was estimated for the first echo and those estimates were applied to the subsequent echoes. All datasets were co-registered to the anatomical MPRAGE image using an affine registration with 12 degrees of freedom (dof). As above, individual echoes were coregistered using the transformation matrix generated from the first echo. The anatomical image was skull-stripped and transformed to Montreal Neurological Institute (MNI) space using a non-linear registration algorithm and segmented into gray matter, white matter, and CSF. The functional data were then transformed to MNI space using the transformation matrix output from the MPRAGE-MNI registration and normalization.

### ASL processing

All ASL signal processing used the first echo with a TE of 9.1 ms. The mean PW signal was calculated by averaging and subtracting the labeled from the control repetitions. A PW timeseries was also generated using the surround subtraction method [49].

### Multi-echo combination and denoising

Multi-echo combination was performed using AFNI. All echoes were used in the combination and were combined using the T2*-weighted approach [38, 50]. First, the voxelwise mean across time of each individual echo dataset was used to estimate the signal immediately after excitation, $\bar{S_{0}}$, and the voxelwise $T_{2}^{*}$, $\bar{T_{2\left(fit\right)}^{*}}$ using log linear regression (Eq 1). Next, the voxewise $\bar{T_{2\left(fit\right)}^{*}}$ was used to determine the weights, $w\left(T_{2}^{*}\right)$ (Eq 2), which were used in a weighted summation of the echoes. In Eqs 1 and 2, *TE*_*n*_ is the n^th^ echo time.

$$S\left(TE_{n}\right)=\bar{S_{0}}\cdot exp\left(-\left(1/\bar{T_{2\left(fit\right)}^{*}}\right)\cdot TE_{n}\right)$$ (1)

$$w\left(T_{2}^{*}\right)=\frac{TE_{n}\cdot exp\left(-TE_{n}/\bar{T_{2\left(fit\right)}^{*}}\right)}{\sum_{n}TE_{n}\cdot \text{exp}\left(-TE_{n}/\bar{T_{2\left(fit\right)}^{*}}\right)}$$ (2)

The multi-echo combined data then underwent an additional denoising procedure using the automated ME-ICA technique and the *meica*.*py* plugin in AFNI [39]. This technique is described in detail elsewhere and classifies independent components as BOLD or non-BOLD based on whether or not their amplitudes are linearly dependent on TE, respectively [39–41]. Components deemed non-BOLD were then regressed out of the multi-echo combined data producing a separate multi-echo combined and denoised dataset. The multi-echo combined and multi-echo combined and denoised datasets were each analyzed separately.

### MEICA performance

To evaluate the performance of the MEICA algorithm for denoising MBME ASL data, the number of components identified, number of components removed, and amount of variance removed by the regression of non-BOLD components were analyzed for each subject. In addition, the mean value of κ, which reflects the goodness of fit to the ΔR2* model, was examined in accepted and rejected components. High values of κ reflect a component that has a strong linear dependence on TE and is likely to be BOLD-related. All components were manually checked to make sure they were correctly classified by the algorithm. This included visually inspecting both the maps of beta-weights, time courses, and Fourier transformed time courses for each component. Components with obvious artifacts and high frequency components were deemed to be non-BOLD.

### rs-fcMRI processing

The preceding steps resulted in four separate datasets for each MBME ASL/BOLD scan that underwent further processing for rs-fcMRI analyses: individual second echo (E2, TE = 25ms), multi-echo combined (MEC), multi-echo combined and denoised (MECDN), and perfusion-weighted (PW) time series. All data were blurred with a 4.5 mm full width at half maximum Gaussian kernel. Several nuisance regressors were removed from the PW, E2, and MEC data including the six rigid-body motion parameters and their derivatives, and white matter and CSF signal. For the E2 and MEC data, the label/control oscillations were regressed out of the data by including a column of alternating -1s and 1s in the design matrix. The E2 and MEC data were also bandpass filtered between 0.01 and 0.1 Hz. The MEC data was fed into the ME-ICA algorithm prior the regression of label/control oscillation and bandpass filtering. Degrees of freedom (dof) were adjusted on an individual subject basis for the MECDN data by subtracting the number of removed components from the dof.

### Resting-state connectivity analysis

Both seed-based and dual-regression analyses were employed for the rs-fcMRI analysis. For the seed-based analysis, several seeds were chosen, including the posterior cingulate cortex (PCC), left (L) and right (R) motor cortex, L/R insula, and L/R hippocampus. Seed locations are shown in Table 1. The PCC seed was chosen to extract the default mode (DMN) network. The insula and hippocampus seeds were chosen based on a previous report [40]. Specifically, the hippocampus seeds were selected to examine the effect of ME-ICA denoising on subcortical connectivity. All seeds were 8 mm radius spheres with the exception of the hippocampus seeds, which were 4 mm radius spheres according to the anatomical structure. The mean signal was extracted from each seed and correlated with each voxel in the brain using Pearson correlation. Correlation values (R) were then transformed to z-scores using a Fisher z-transform. A group analysis was then conducted using a one-sample t-test on the Fisher-transformed z-scores. For individual and group maps, voxels with corrected p<0.005 (minimum cluster size = 46, cluster corrected α = 0.05) were considered significant. A cluster-corrected α of 0.05 means the probability of getting a single noise-only cluster is 0.05.

**Table 1:** Montreal Neurological Institute coordinates of seed regions used in the functional connectivity analysis.

Seed	x	y	z
PCC^a^	0	-54	26
MFG^a^	6	56	14
L Motor^a^	-34	-24	60
R Motor^a^	40	-18	56
L Insula^a^	-40	-8	8
R Insula^a^	40	-8	8
L Hippocampus^b^	-28	-28	-12
R Hippocampus^b^	24	-28	-12

Abbreviations: PCC = posterior cingulate cortex; MFG = medial frontal gyrus; L = left; R = right.

^a^8mm radius spherical ROI.

^b^4mm radius spherical ROI

In addition to the seed-based analysis, a dual regression analysis was performed to compare across datasets (i.e., E2, MEC, MECDN, PW) [51] and implemented in FSL. For this analysis, a template containing predefined ICA components of interest was used [52]. The template contains 7 spatially independent networks derived from a clustering approach, which identified networks of functionally coupled regions using data from 1000 normal subjects [52]. For each component of interest, the procedure described in [53] was applied. First, the spatial component map was used as a spatial regressor in a general linear model (GLM) and the temporal signal associated with the network was extracted. This signal was then used as a regressor in a second GLM to find the subject-specific spatial maps associated with the template network. Group maps were created for each dataset by converting correlations to z-scores and using a one-sample t-test.

### CBF/BOLD coupling

An additional analysis was carried out to examine the coupling of the CBF and BOLD signals for each of the datasets. The CBF/BOLD coupling was assessed by correlating the signals from each of the E2, MEC, and MECDN datasets to the PW data on a voxelwise basis using Pearson correlation. Signal was extracted from the datasets following rs-fcMRI processing. Correlation maps were converted to z-scores using a Fisher’s z transform, and a group t-test was performed across subjects for each dataset to create group CBF/BOLD coupling maps. The CBF/BOLD coupling maps were thresholded at p<0.005 (minimum cluster size = 46, cluster corrected α = 0.05).

### Quantitative analysis

Whole-brain tSNR was computed for the timeseries datasets prior to detrending and nuisance regression and defined as the mean signal divided by the standard deviation across the timeseries. For the PW data, tSNR was calculated in GM only. In addition, the mean correlation value was extracted from significantly correlated voxels for each seed region of interest (ROI). This was computed using two masks. First, a connectivity mask was determined for each subject, seed ROI, and dataset by thresholding at a corrected p<0.005 (minimum cluster size = 46, cluster corrected α = 0.05). The number of correlated voxels was also computed using this mask. Second, using voxels that were significantly correlated for all four datasets with corrected p<0.005 (minimum cluster size = 46, cluster corrected α = 0.05), an overlap mask was created for each ROI and subject. Metrics, including tSNR, mean correlation, and network size, were compared using a paired t-test. Comparisons with a Bonferroni corrected p-value < 0.05 were considered significant.

## Results

**Fig 3:**
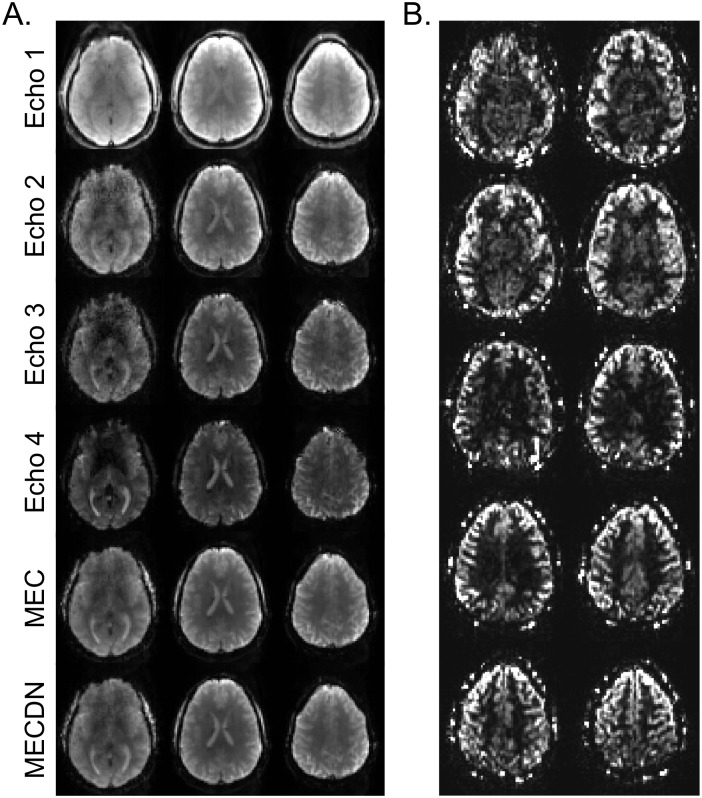
Representative perfusion-weighted, individual echo, and multi-echo images. (A) Example individual echo, MEC, and MECDN images from one subject. Image SNR decreases with echo time. Image quality improves with echo combination and signal in the inferior portions of the brain is recovered. All images share the same color scale. (B) Perfusion weighted images. Images were produced by averaging and subtracting the label images from the control images. High-resolution whole-brain images were collected in a relatively short amount of time with reduced signal loss caused by T1-relaxtion of the labeled blood.

Representative individual echo, MEC, MECDN, and mean PW images are shown in Fig 3. As expected, SNR decreases with TE. Image quality is similar for the MEC and MECDN data and improved over the individual echo cases. tSNR was significantly higher for the MECDN data (106 +/- 11) compared to the MEC (69 +/- 12) and E2 data (47 +/- 9) and for the MEC data compared to the E2 data (MECDN > E2, P < 0.0001; MECDN > MEC, P = 0.0009; MEC > E2, P = 0.005).

### MEICA performance

Table 2 shows summary statistics for the ME-ICA analysis. On average, approximately 64 components were identified, 23 of those were removed accounting for 79% of the variance, As expected, the mean κ in accepted components was higher than in rejected components.

**Table 2:** MEICA performance.

Subject #	# of Components Identified	# of Components Removed	Variance Removed	Mean κ Accepted Components	Mean κ Rejected Components
**1**	40	17	86.2	33.6	8.3
**2**	66	20	74.7	31.0	9.1
**3**	73	27	84.9	21.5	8.1
**4**	73	25	73.6	28.2	8.8
**5**	52	24	86.2	27.5	10.5
**6**	72	29	80.7	37.8	11.3
**7**	70	18	63.7	32.2	9.8
**Mean (Stdev)**	63.7 (12.8)	22.9 (4.6)	78.6 (8.4)	30.2 (5.2)	9.4 (1.2)

**Fig 4:**
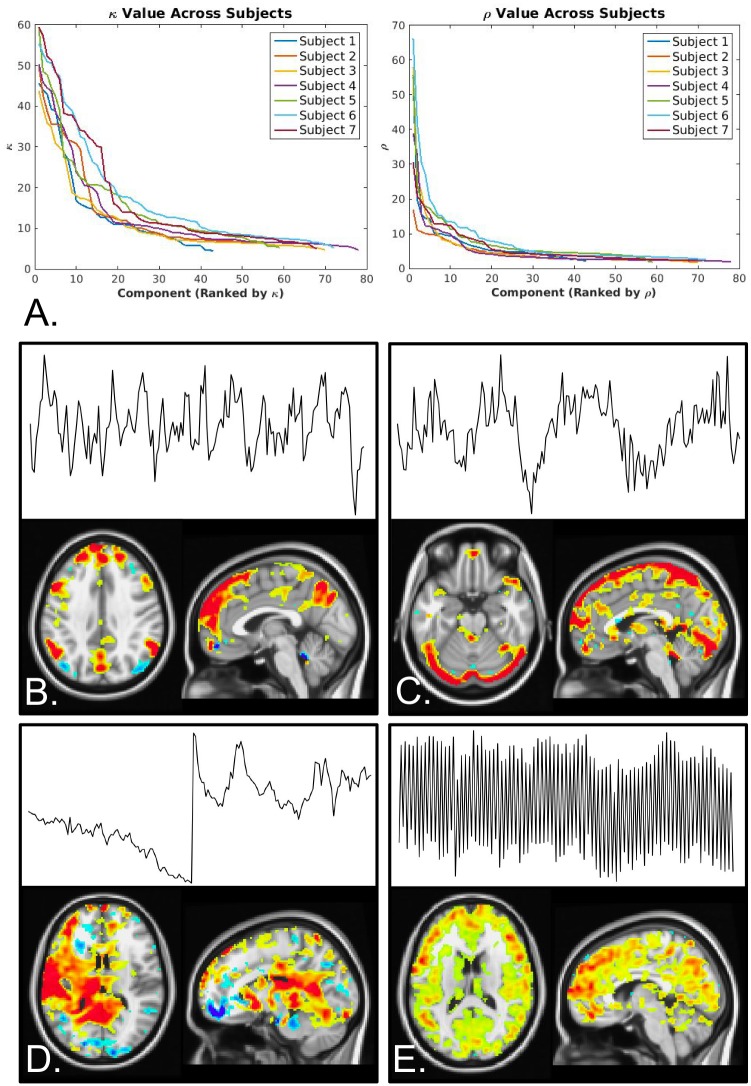
MEICA performance. A. Curves of κ and ρ for all subjects. Both curves display the characteristic “L” shape expected from the ME-ICA algorithm. κ describes the goodness of fit to the TE dependence of each component and ρ described the fit to a ΔS_0_ model. In general, components with κ above and ρ below the elbow are kept in the denoised timeseries. B-E display example networks from one representative subject. B. An example accepted BOLD component (DMN). C. A rejected component classified as an R2* artifact. D. A rejected non-BOLD component. E. A rejected PW component.

Fig 4A shows plots of κ and ρ verses component ranked by κ and ρ respectively. Here, ρ is a measure of how well a component fits the ΔS_0_ model. In contrast to κ, high ρ indicates components are likely artifacts. Both plots show the characteristic “L” shape described in Kundu et al. [40] for all subjects. All accepted components were correctly classified as BOLD-related networks. These components and associated beta-weight maps were free from obvious artifact, and in most cases were closely matched to well-known networks from the literature (i.e. DMN, motor network, salience network, etc.). Examples of accepted and rejected networks are shown in Fig 4(B)–4(E). Fig 4B shows an accepted network, the DMN. Fig 4C and 4D shows networks rejected due to being an R2* artifact and non-BOLD component respectively. Fig 4E shows a rejected perfusion-weighted component, characterized by oscillating signal. In fact, a PW signal was identified for each subject and correctly rejected as non-BOLD by the algorithm.

### Functional connectivity

**Fig 5:**
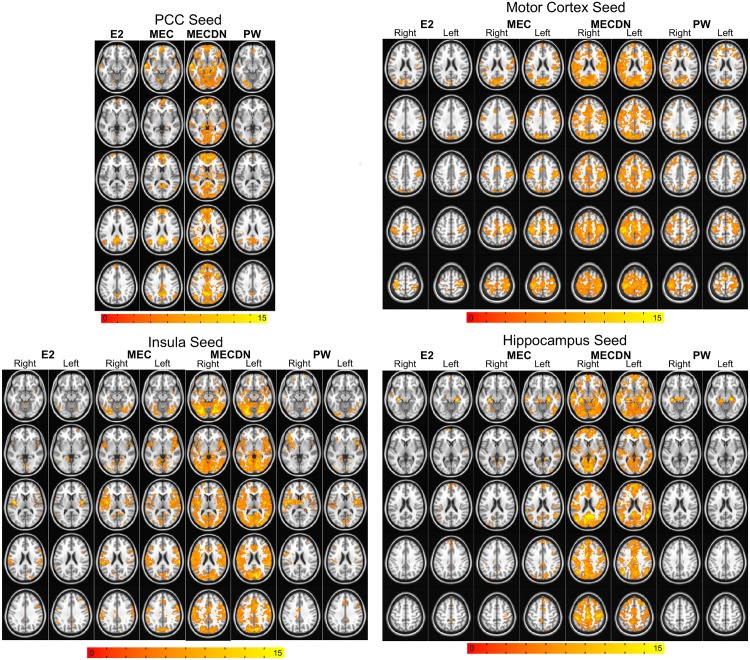
Group seed-based functional connectivity maps. Connectivity maps are displayed for PCC, L/R motor cortex, L/R insula, and L/R hippocampus seeds. Maps are the result of a one-sample t-test on the Fisher-transformed z-scores and were thresholded at P < 0.005 with minimum cluster size = 46, α = 0.05. For all seed regions, connectivity was markedly increased in terms of network size and correlation strength for the MECDN datasets compared to the others. Limited insula and hippocampus connectivity was observed for the E2 and PW datasets. Bilateral insular connectivity was seen for the MEC dataset. The MECDN data produced significant bilateral connectivity with long-range connections for both the insula and hippocampus seeds. Robust connectivity was detected with the PW data for the PCC and motor network seeds. Some bilateral connectivity was seen for the insula seeds.

Resting-state networks were extracted using seeds in the PCC, L/R motor cortex, L/R insula, and L/R hippocampus for the E2, MEC, MECDN, and PW datasets. Fig 5 shows the seed-based functional connectivity results from the group analysis across subjects for each seed with a cluster-size corrected threshold of p<0.005. For the E2 data, some bilateral and long-range connectivity was seen for the PCC and motor cortex seeds. Little connectivity was seen away from the seed region for the insula and hippocampus seeds. An increase in the number of clusters and cluster size and strength were observed for the MEC data. A further increase in the number of clusters was seen for the MECDN data and similar clusters tended to be larger and have stronger correlations. For the insula seeds, significant bilateral and long-range connectivity was observed for the MECDN data, which included strong connectivity in the temporal lobe. For the hippocampus seeds, minimal connectivity was seen for the E2 and MEC datasets. For the MECDN dataset, connectivity was detected with the contralateral hippocampus, areas associated with the DMN including PCC, MFG, middle temporal gyrus, and parietal cortex. Even at the relatively high threshold, robust bilateral, long range PW data connectivity was observed for the PCC and motor cortex seeds. More-limited connectivity was seen for the insula and hippocampus seeds, although some long-range connections were observed for the insula seeds.

**Fig 6:**
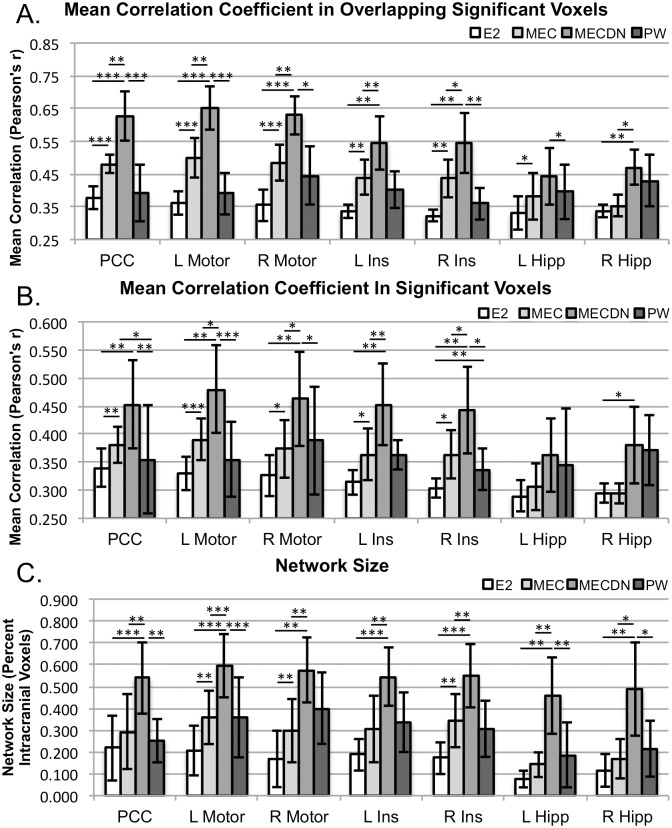
Quantitative results. Mean correlation in overlapping significant voxels (Top), mean correlation in significant voxels for the E2, MEC, MECDN, and PW data separately (Middle), and network size, displayed as a fraction of intracranial voxels (Bottom). Voxels with P < 0.005 and minimum cluster size = 46, α = 0.05 were considered significant. Parameters were extracted on an individual subject basis. * = P < 0.05; ** = P < 0.01; *** = P < 0.001, Bonferroni-corrected.

Quantitative results are shown in Fig 6. Mean correlation for both the overlap maps and individual maps was significantly increased for the MEC data compared to the E2 data, and for the MECDN data compared to both the MEC and E2 data for the PCC, L/R motor, and L/R insula with the exception of MECDN vs. MEC for the PCC using individual masks. Some significant differences were seen for the hippocampus seeds. The MECDN correlation was significantly higher than the PW correlation for the PCC, L/R motor, and R insula seeds for both masks. Similar trends were observed for the network size, displayed as a fraction of intracranial voxels. Of note, the MECDN had a significantly higher network size compared to the MEC and E2 data for the L/R hippocampus seeds. In addition, network size was higher for the PW data compared to the E2 data for the L/R motor cortex, and R insula seeds.

**Fig 7:**
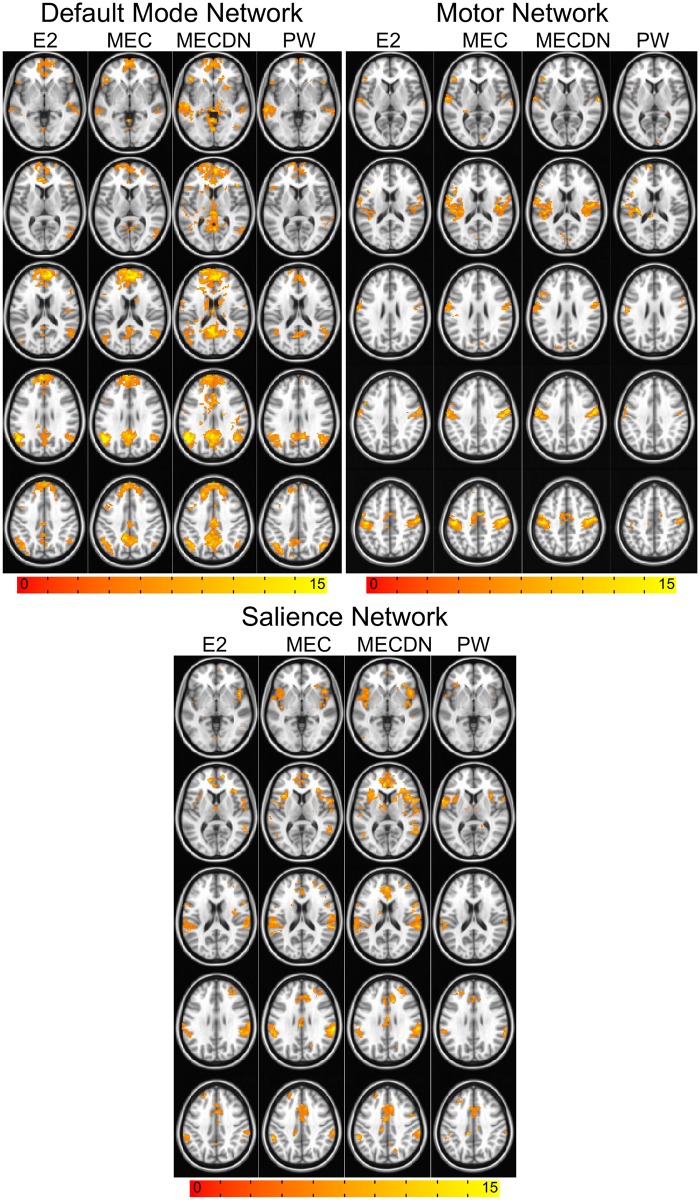
Group dual-regression based functional connectivity maps. Connectivity maps are displayed for the default mode network, motor network, and salience network. Maps are the result of a one-sample t-test on the z-scores and were thresholded at P < 0.005 with minimum cluster size = 46, α = 0.05. Additional clusters were seen for the MEC data compared to the E2 data and for all networks, and for the MECDN data compared to the MEC data for the DMN and salience networks. Existing clusters also tended to be larger for the MECDN data for these networks. Motor network connectivity was similar between the MEC and MECDN datasets. Bilateral, long range connectivity was seen for all datasets, including the PW data.

A dual regression approach was also incorporated to assess functional connectivity. All seven networks were analyzed, but three representative networks, the DMN, motor network, and salience network, are shown in Fig 7. These networks were chosen to match the networks extracted from the seed based analysis. For all 7 networks, the MEC data had stronger and more widespread connectivity compared to the E2 data. The MECDN data had more widespread connectivity compared to the MEC data for the DMN and salience networks and similar connectivity for the motor network. This was also consistent across all networks as MECDN data had similar or stronger connectivity compared to the MEC data. The PW connectivity was not as strong as the BOLD connectivity, though bilateral connectivity was observed for most networks.

### CBF/BOLD coupling

**Fig 8:**
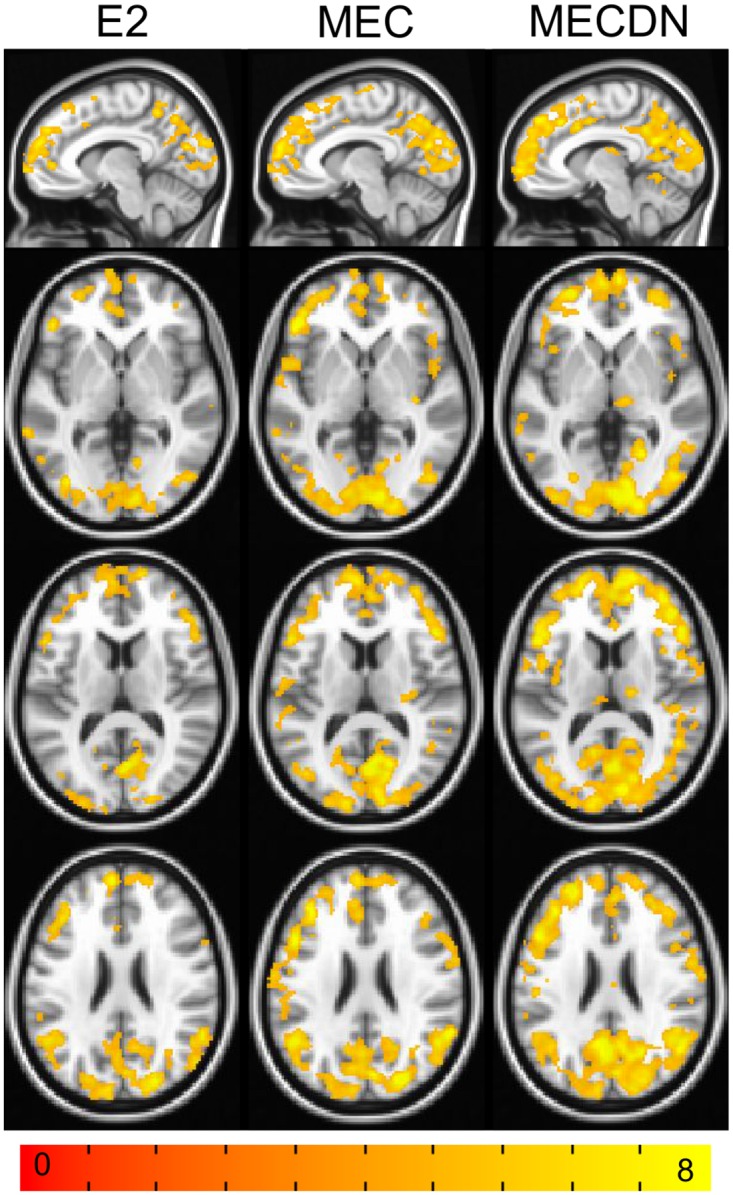
Group CBF/BOLD coupling. Results are shown for the E2, MEC and MECDN datasets. Widespread coupling was observed and increased coupling was seen within well-known brain networks including the DMN, and visual networks. Stronger, more widespread coupling was seen for the MECDN data compared to the MEC and E2 data.

Results of the CBF/BOLD coupling analysis are shown in Fig 8, which depicts the group CBF/BOLD coupling for the E2, MEC, and MECDN datasets. The coupling was widespread, but was strongest in the DMN and the visual network. Correlation strength and area were increased for the MECDN data compared to the MEC and E2 data.

## Discussion

In this study, an MBME ASL/BOLD sequence was implemented to evaluate resting-state connectivity. This sequence allows for the simultaneous collection of high spatial resolution ASL and BOLD-weighted time series. Four total echoes were collected, which enabled the use of ME-ICA denoising to improve data quality. Resting-state fcMRI data were collected using this sequence and resting-state networks were compared between MEC, MECDN, and the E2 datasets collected in a typical simultaneous ASL/BOLD experiment. Networks extracted using the PW signal were also examined. MECDN data had significantly increased tSNR compared to MEC and E2 data. In addition, the results of a seed-based analysis showed that resting-state brain networks were larger and connections were stronger for the MECDN data. A dual regression analysis was performed and showed additional clusters for the MECDN data. A CBF/BOLD coupling analysis revealed increased CBF/BOLD coupling in resting-state brain networks when the data were processed using ME-ICA denoising.

The MBME ASL sequence has several advantages over other sequences. Previous simultaneous ASL/BOLD studies were limited in the number of slices that could be acquired. Because of the addition of multiband imaging, whole-brain simultaneous ASL/BOLD data can be acquired with MBME ASL with limited T1-relaxation of the tagged blood due to reduced interslice labeling delay times and total readout times. Thus, high quality, whole-brain PW data can be obtained. Another advantage is the ability to collect additional echoes beyond the two echoes used in typical simultaneous ASL/BOLD sequences. The additional echoes can be combined to increase tSNR for the BOLD echoes with a small increase in TR. Furthermore, ME-ICA denoising can be applied to automatically remove artifactual and non-BOLD components from the data [39–41].

The performance of the MEICA algorithm was investigated. The algorithm performed well for all subjects. The mean number of rejected components was 22.9 +/- 4.6 representing 78.6 +/- 8.4% of the normalized variance. There were no misclassified components observed, and for all subjects tSNR for the MECDN data was increased compared to the E2 and MEC data. In addition, all subjects had κ and ρ curves (Fig 4A) with the expected “L” shape [40]. The κ and ρ thresholds are set by finding the elbow of this curve. In general, components with κ above the κ threshold are classified as BOLD components while components with ρ above the ρ threshold are classified as artifacts. Interestingly, a perfusion related component was found for all subjects (Fig 4E). This component was characterized by oscillating signal caused by the signal differences between label and control acquisitions. In all cases this component was correctly classified as non-BOLD and removed from the data. Thus, label/control oscillations did not have to be removed from the data prior to ME-ICA (either by filtering or adding a column of -1s and 1s to the design matrix for regression), or explicitly removed from the denoised data after ME-ICA.

ME-ICA denoising led to increased resting-state network area and strength. In order to better visualize the benefits of the multi-echo acquisition, a relatively high threshold of p<0.005 was used for all voxel-wise analyses. For the seed-based analysis, at this threshold, connectivity was limited, but present for the E2 data. Additional clusters were observed for the MEC and MECDN data. The MECDN clusters were larger and connections stronger than those in the MEC data, particularly in the anterior portions of the brain. More widespread motor network connectivity was observed for the MECDN data, including in the insula, which has been shown to be part of the somatomotor network [54, 55].

Bilateral insular connectivity was seen for the MEC data; however, more widespread bilateral connectivity was observed for the MECDN data, which included clusters in the DMN regions, motor cortices, and occipital lobe. The insula is known to be involved in a number of processes, including motor and visual processing, and has shown connectivity with the DMN in previous studies [56, 57]. Hippocampal connectivity was limited for the MEC data. Calculating subcortical connectivity is difficult because CSF and blood flow pulsation lead to reduced BOLD contrast. However, for the MECDN data, bilateral connectivity was observed and clusters were seen in the PCC, anterior brain regions, middle temporal gyrus, and motor cortices. These results mirror the results from Kundu et al. in their seminal paper describing ME-ICA [40]. They found very little subcortical connectivity using a T2*-weighted echo combination, bandpass filtering, and typical nuisance signal regression. When ME-ICA was employed, significant hippocampal connectivity was found with sensory, temporal, and premotor cortical areas.

The relatively weak connectivity for the seed-based E2 case is likely due to a combination of the stringent threshold (P<0.005), the relatively small number of subjects (7), and the relatively long TR (3.5s). Of note, however, is that by collecting more than 2 echoes ME-ICA denoising can be used to compensate for these shortcomings. Additional echoes do cause a slight increase in TR, however the majority of the TR in a pCASL acquisition is the labeling (1.5s) and PLD (1.0s). Therefore, this effect is limited. Fig 5 shows extensive connectivity for all seeds for the MECDN case even at high thresholds.

Fig 6C shows the percent of significant intracranial voxels for the seed-based analysis. For the E2 case, over 20% of voxels were significantly correlated with the PCC and motor seeds, while Fig 5 shows much more limited connectivity. This discrepancy is a result of the voxel counts in Fig 6 being extracted on a per-subject basis using Pearson correlation and Fig 5 being the result of a 1-sample t-test across a relatively small number of subjects using relatively high threshold (P<0.005). Many more voxels reached significance on an individual subject basis compared to across subjects.

Using the dual regression analysis, stronger, more widespread connectivity was observed for the MEC data compared to the E2 data for all networks. Larger clusters were observed for the MECDN data in the DMN in the anterior cingulate cortex (ACC) inferior PCC, and the thalamus. In the salience network, larger cluster sizes were observed in the insula and ACC for the MECDN data compared to the MEC data. MECDN and MEC connectivity was very similar for the motor network.

The increase in connectivity for MECDN data is likely due to the increase in tSNR. Murphy et al. showed the relationship between tSNR and the number of time points necessary to detect activation for a certain effect size for BOLD data [58]. This relationship is nonlinear, and a relatively small increase in tSNR can result in a large reduction in the number of time points necessary to detect significance. The use of ME-ICA denoising in this study resulted in a tSNR increase from 47 for E2 data to 106 for the MECDN data. This greatly increases statistical power, reducing the number of time points necessary to detect significance. Thus, the long TR required in ASL, and subsequent reduced number of observations in a given period of time, is less problematic with this method.

We were also able to consistently detect resting-state networks using the PW timeseries, especially for the seed-based analysis. For example, bilateral connectivity was seen for the PCC, motor cortex, and insula seeds, despite the high threshold. This is quantified in Fig 6, where correlation strength and network size were similar or greater for the PW data compared to both the MEC and E2 data. The use of multiband imaging with a blipped-CAIPI allowed for the collection of whole-brain high-resolution data with limited SNR losses due to g-factor considerations. Furthermore, MB imaging allowed T1-related PW signal loss to be minimized.

To explore one possible use for the simultaneous ASL/BOLD sequence, we analyzed CBF/BOLD coupling, defined as the correlation between the PW and BOLD timeseries. We found CBF/BOLD coupling was widespread and variable, however, coupling was increased in well-known brain networks including the DMN and visual network. These results support the results from Tak et al. [16]. They also found increased positive coupling in the DMN and visual network. In addition, they found increased coupling in the task-positive network including the intraparietal sulcus (IPS), dorsal anterior cingulate cortex (dACC), and middle temporal region (MT). We did see significant coupling in this region for the MEC and MECDN datasets, but not to the extent they showed. One interesting finding was coupling strength and area were stronger for the MECDN data than for the MEC and E2 data. This likely stems from a reduction in noise for the MECDN data, leading to more accurate connectivity and coupling results. This novel sequence could be a valuable tool for studies on neurovascular coupling [59–61].

This study had some limitations. First, the number of subjects was relatively small. The purpose of this study was to determine the feasibility of using the MBME ASL/BOLD sequence to detect resting-state connectivity; thus, a small subject size was justified. Significant group networks were still identified. Second, a relatively short PLD was employed for this study. This could be an issue for interleaved and MB slice acquisitions, where superior slices are acquired earlier in the readout, and could lead to intravascular artifacts. Despite this, we were able to robustly detect brain networks using ASL. Other studies have employed short PLDs to study functional connectivity. One study used a 3D pCASL acquisition and PLD = 0.600s to perform a functional connectivity analysis [62]. They further used ICA to extract and remove intravascular signal. This method could be utilized in future studies with MBME ASL. Another study analyzed functional connectivity with both a 3D pCASL sequence with PLD = 1.0s and a BOLD EPI sequence [63]. They found robust ASL-based connectivity and considerable overlap between ASL and BOLD networks. Future studies should examine the effect of PLD on rs-fcMRI for MB pCASL scans. Background suppression, in which the background signal is reduced using saturation and inversion pulses, has been shown to increase tSNR and improve the sensitivity of ASL [12, 64]. However, the BOLD signal is drawn from the background signal. Thus, reducing the background signal will reduce BOLD SNR and tSNR. In spite of this, one recent study recommended background suppression for 2D dual-echo ASL acquisitions [13] finding the large CBF signal gains offset the slight BOLD sensitivity losses. The additional echoes in MBME ASL/BOLD may also help offset some of the BOLD SNR losses. Further studies are needed to determine the effects of BS on MBME ASL/BOLD acquisitions. Finally, we did not collect heartbeat or respiration measurements as part of the functional acquisitions. Thus, respiration and heartbeat signals were not regressed out of the data. For the MEDN data, the MEICA denoising process should identify and remove the non-BOLD physiological signals as noise. However, physiological noise in fMRI can come from either BOLD or non-BOLD sources. Future studies should examine the effect of regressing physiologic variables from MBME ASL data.

This sequence has several other potential applications. In addition to assessing the contributions of CBF to the BOLD response using CBF/BOLD coupling [15, 16], this sequence can be used to measure CVR, CMRO_2_ [19, 22], and CBF-CMRO_2_ coupling [21, 59–61, 65]. The latter has also been used to investigate the effects of drugs [66] and hypercapnia [67] on brain physiology. By incorporating MB imaging and more than two echoes, our sequence can provide high-resolution, whole-brain images with increased BOLD sensitivity.

In conclusion, MB, ME, pCASL, and BOLD imaging were combined into one sequence. This sequence allowed for the simultaneous acquisition of high spatial resolution ASL and BOLD timeseries. Functional connectivity was robustly detected using ASL and BOLD datasets. In addition, the collection of more than two echoes allowed for MEICA denoising to be applied. This technique resulted in the detection of larger, stronger resting-state networks, increased CBF/BOLD coupling, and increased signal stability.

## Abbreviations

ASL: arterial spin labeling
BOLD: blood oxygenation dependent
CBF: cerebral blood flow
DMN: default mode network
E2: individual second echo (TE = 25ms)
MB: multiband
MBME: Multband, multi-echo
MEC: multi-echo combined
MECDN: multi-echo combined and denoised
ME-ICA: multi-echo independent component analysis
pCASL: pseudo-continuous arterial spin labeling
PW: perfusion-weighted
rs-fcMRI: resting state functional connectivity MRI
